# The role of vessel wall physiology in predicting coronary bypass graft patency

**DOI:** 10.1186/1749-8090-1-5

**Published:** 2006-03-03

**Authors:** Steve K Singh, Stephen E Fremes

**Affiliations:** 1 Division of Cardiac and Vascular Surgery, Sunnybrook and Women's Health Sciences Centre, 2075 Bayview Avenue, Suite H-410, Toronto, Ontario, M4N 3M5, Canada; 2 University of Toronto, Toronto, Canada

## Abstract

Not applicable.

## Text

The phase one results from the randomized Radial artery versus Saphenous Vein Patency (RSVP) trial are published in the current issue of the *Journal *[1]. Fifty-two patients had quantitative angiography with Thrombolysis in Myocardial Infarction (TIMI) flow assessment, approximately 3 months after coronary bypass graft surgery. A subset of 7 patients had *in-vivo *assessment of graft flow and vessel wall physiology, using intravascular Doppler ultrasound and pharmacologic manipulation. The investigators found that 11% of radial artery (RA) grafts had either a greater than 50% stenosis or diffuse narrowing (string sign), no RA was completely occluded, 5% of long-saphenous veins (LSV) were completely occluded, and the remaining RA or LSV grafts were perfectly patent. RA grafts were found to have intact endothelial function at 3 months post-operatively, whereas LSV grafts did not. Although flow volumes were similar in both conduits, the size match of conduit-to-target vessel was better with RA grafts. Hence, the investigators concluded that the RA is a more physiologic conduit for coronary bypass surgery.

The natural history of coronary bypass grafts suggests that biologic properties of the conduit vessel strongly influence graft function and patency, and the intact physiology of the endothelium is deemed to be a primary determinant [2-4]. The article in the *Journal *by Chong *et al *corroborates the concept that early venous graft failure primarily involves complete occlusion, presumed to be related to thrombosis [1,4]. In contrast, early RA failure, which is comparable in frequency, is a consequence of diffuse narrowing (string signs) [1,5]. The latter is related to the vessel wall properties innate to arterial conduits. The endothelium produces potent vasodilators, antithrombotic and antiatherogenic properties. These are known to be released in greater amounts in arterial than venous conduits [3,6].

Since physiology is maintained in RAs, the string sign may be a clinically adaptive response to optimize flow characteristics. These grafts respond to endogenous (and exogenous) vasodilators to alter blood flow in high demand situations and also optimize the graft size-match with the target vessel. This balance between endothelium-mediated vasoconstriction and vasodilation was revealed by the investigators in their study. They demonstrated autoregulation of the RA conduit diameter, which adapted to match target vessel size [1]. Also, the investigators demonstrated that endothelial-dependent vasodilation is preserved in the RA at 3-months post-surgery, but not with the LSV grafts, such that administration of intravenous nitrate in RA patients with string signs resulted in all having some degree of dilation [1].

However, maintaining vessel wall physiology may also lead to maladaptive results. The proclivity of arterial conduits to spasm is an example of sensitivity to endogenous and exogenous vasoconstrictors. A sub-group analysis of RA patients from the Radial Artery Patency Study (RAPS) found that use of peri-operative vasoconstrictors (alpha adrenergic agonists) was an independent predictor of RA string sign [5,7]. Calcium channel blockers are often employed post-operatively as an anti-spasmodic therapy. However, their efficacy and the duration of treatment remain controversial [8].

Chong *et al *also showed that flow volumes were the same in both RA and LSV grafts [1]. This emphasizes the influence of competitive flow on graft patency. Specifically, it has been shown that target vessel stenosis less than 90% was an independent predictor of RA graft failure (string sign) [7]. This sensitivity, again is a consequence of a functional vessel wall responding to poor flow conditions.

There are certainly advantages and disadvantages to the RA in having preserved vessel wall physiology. There is evidence that string signs (in both RA and IMAs) are transient and do reverse, when studied using serial angiographic imaging [9]. Nonetheless, the most relevant outcome is post-operative patient clinical status. This does not seem to be impacted; no difference in myocardial infarction or angina at 3 months and 12 months has been reported in patients who developed a string sign versus those who did not [10]. Angina occurred in a small group of patients and was related to TIMI 1 flow. The majority of patients with string signs still had greater than TIMI 2 flow and were asymptomatic.

With appropriate harvesting, target vessel selection and peri-operative medical management, the radial artery provides a more physiologically appropriate conduit with functionally normal vessel wall behaviors, when compared to a saphenous vein. The string sign may in many cases be an adaptive consequence of RAs and without clinical consequences. There seem to be no pathological evidence in RAs that show string sign, nor does it seem to be a permanent or clinically significant phenomenon. Using similar measures as Chong *et al*, Possati *et al *have shown that endothelial function remains preserved in the RA at 10-years post-operation [11]. What remains to be seen is if normal vessel physiology will result in improved long-term graft patency by avoiding the process of accelerated atherosclerosis, which is prevalent in venous graft and often occurs secondary to endothelial damage or dysfunction.

## Competing interests

The author(s) declare that they have no competing interests.

## Authors' contributions

SKS and SEF were both equally involved in the conceptualization, research, writing and manuscript preparation of this editorial.
